# A *Begomovirus solanumdelhiense* Vector for Virus-Induced Gene Silencing in Melon

**DOI:** 10.3390/pathogens14121269

**Published:** 2025-12-10

**Authors:** Yufei Han, Qiansheng Liao, Ping Gao, Liqing Zhang, Bingqian Wan, Lihui Xu, Shigang Gao, Zhiwei Song, Fuming Dai, Rong Zeng

**Affiliations:** 1 College of Fisheries and Life Sciences, Shanghai Ocean University, Shanghai 201306, China; hanyf@163.com; 2 College of Life Sciences and Medicine, Zhejiang Sci-Tech University, Hangzhou 310018, China; qshliao@aliyun.com; 3 Eco-Environmental Protection Research Institute, Shanghai Academy of Agricultural Sciences, Shanghai 201403, China; gaoping@saas.sh.cn (P.G.); zhangliqing@saas.sh.cn (L.Z.); bingqian@saas.sh.cn (B.W.); xulihui@saas.sh.cn (L.X.); gaoshigang@saas.sh.cn (S.G.); songzhiwei061@163.com (Z.S.)

**Keywords:** melon, *Begomovirus solanumdelhiense*, virus-induced gene silencing, *Phytoene desaturase*

## Abstract

In this study, the insert length, location within the coat protein-encoding gene, and sequence orientation of the target fragment were optimized to construct an efficient virus-induced gene silencing (VIGS) system in melon using a *Begomovirus solanumdelhiense* vector. Existing systems are mostly RNA viruses, requiring in vitro synthesis of viral strands that are prone to degradation, although they exhibit high infectivity and stability in cucurbit hosts and ease of manipulation. This vector was selected for its more stable genome structure and these advantages. The melon phytoene desaturase (CmPDS), a key gene of carotenoid biosynthesis, was selected as a reporter gene to evaluate the effects of the VIGS system. Our results revealed that the melon leaves in all the VIGS treatments exhibited a typical photobleaching phenotype at 21 days post-inoculation. Moreover, reverse transcription quantitative real-time PCR revealed a significant reduction in the mRNA levels of PDS in melon. The highest silencing efficiency (lowest PDS mRNA levels) was achieved by the VIGS vector harboring a 165 bp CmPDS fragment at the 3′ end of the AV1. These findings not only establish a more efficient VIGS protocol for melon but also provide a foundation for developing novel virus-based silencing tools applicable to functional genomics and cucurbit crop improvement, particularly for traits requiring precise gene expression modulation such as disease resistance and fruit quality.

## 1. Introduction

Melon (*Cucumis melo* L.) is a globally significant economic crop. The validation of gene functions in melon relies primarily on stable genetic transformation systems. However, genetic transformation efficiency in melons is significantly hindered by genotype-specific constraints, which severely restricts the application of breeding strategies. Virus-induced gene silencing (VIGS), a reverse genetics tool based on the plant’s antiviral mechanisms, has been widely applied in plant gene functional studies, because of its independence from plant genetic transformation systems, short experimental cycles, simplicity of operation, and ability to silence single gene or gene family members [1,2,3]. Recently, it has been successfully applied to research on plant-directed evolution, further demonstrating its potential functionality [4]. Its principle involves the insertion of specific fragments of target genes into viral vectors, the use of viral replication and transmission capabilities to induce the silencing of endogenous genes in plants, and the inference of gene functions through resulting phenotypic or physiological changes [5]. Currently, VIGS vectors for cucurbit crops rely primarily on RNA viruses, such as tobacco rattle virus (TRV) [6], tobacco ringspot virus (TRSV) [7], and cucumber green mottle mosaic virus (CGMMV) [8]. Among these, the silencing efficiency, infection range, and stability of VIGS vectors constructed using TRV are particularly outstanding, making them the most widely applied tools for gene silencing [9,10,11]. However, the use of RNA viruses as VIGS vectors is limited because of their inherent structural instability and susceptibility to degradation during in vitro synthesis.

Compared to RNA viral vectors such as TRV, DNA viral vectors offer distinct advantages: they do not require in vitro transcription to synthesize RNA viral chains, are more stable, and are less prone to degradation during carrier preservation than RNA vectors are. Additionally, compared to RNA genomes, DNA genomes are inherently more stable, have lower mutation rates, and ensure higher fidelity of foreign-expressed products [12]. Currently, the majority of research efforts on engineering DNA viruses as VIGS vectors have focused on geminiviruses. Geminivirus-based vectors exhibit a rapid replication capacity for exogenous small double-stranded RNA (dsRNA) fragments, enabling efficient gene silencing. Notably, their modular genomic components are amenable to genetic engineering, making these viruses an ideal platform for VIGS applications. Multiple geminiviruses, including abutilon mosaic virus (AbMV), African cassava mosaic virus (ACMV), tomato yellow leaf curl China virus (TYLCCNV), cotton leaf crumple virus (CLCrV), tomato golden mosaic virus (TGMV), bhendi yellow vein mosaic virus (BYVMV), and cabbage leaf curl virus (CaLCV) [13,14,15,16,17,18,19], have been successfully employed as VIGS vectors to silence endogenous genes in host plants, demonstrating their robust functional utility in plant molecular biology studies. However, current research has focused primarily on model plants such as *Nicotiana benthamiana*. However, its application in cucurbits still faces key challenges: weak infectivity of viral vectors, complex vascular systems that may impede viral movement, and transient silencing effects (typically weeks).

*Begomovirus solanumdelhiense* (formerly known as tomato leaf curl New Delhi virus, ToLCNDV), a bipartite single-stranded DNA virus belonging to the Geminiviridae family, genus Bean golden yellow mosaic virus [20,21,22], consists of two circular DNA components (DNA-A and DNA-B) that range from 2600 to 2800 nucleotides. DNA-A encodes viral replication-related proteins, including those required for replication, gene expression regulation, and capsid formation. The coat protein (CP), encoded by the *AV1*, is responsible for assembling the viral capsid to protect the viral genomic DNA. Due to its non-essential and stable capacity to accommodate foreign gene fragments, the *AV1* has become an ideal insertion site. The open reading frame (ORF) encoded by DNA-B is associated with viral movement within and between host plant cells [23,24,25], enabling efficient systemic infection capacity. ToLCNDV has a broad host range and is highly infectious and can infect various crops (e.g., *Solanaceae* and *Cucurbitaceae*), ornamental plants (e.g., *Chrysanthemum*)*,* weeds, and wild plants [26,27,28,29]. Phytoene desaturase (PDS) is a key enzyme in the carotenoid biosynthesis pathway. It is commonly used as an indicator gene in VIGS studies because of its silencing-induced photobleaching phenotype in plant leaves caused by carotenoid deficiency [30,31,32]. This study was based on a full-length DNA infectious clone of ToLCNDV, in which melon plants were used as the experimental material and the *phytoene desaturase CmPDS* was used as an indicator. The objective of this study was to systematically evaluate the effects of the length institution site with the *AV1*, and sequence orientation of the target fragment on the silencing efficiency of the ToLCNDV-mediated VIGS system. By optimizing these parameters of the target gene, this study aimed to establish an efficient VIGS technical system tailored for melon.

## 2. Materials and Methods

### 2.1. Plant, Inoculation, and Host Bacterial Materials

Melon (Tinglin) seeds were soaked in 50 °C sterile water for 3–4 h, then placed in Petri plates containing wetted filter cotton gauze in darkness at 25 °C. Germinated seeds were transplanted into pots with nutrient matrix and grown in a 25 °C growth chamber under a 16 h light/8 h dark cycle. Melon seedlings, cultivated for approximately one week (only with two cotyledons), were used for inoculation.

pCambia-ToLCNDV-1.3A and pCambia-ToLCNDV-1.6B were the infectious clones of ToLCNDV DNA-A and DNA-B (Figure S1), respectively, and were used for ToLCNDV infection of melon by *Agrobacterium*-mediated inoculation [11].

*Escherichia coli* strain T1 and *Agrobacterium tumefaciens* strain GV3101 were purchased from Shanghai WEIDI Biotech Co., Ltd. (Shanghai, China).

### 2.2. Design of Target Fragments and Construction of Empty Vectors

The PDS sequence of *Cucumis melo* was aligned with those of *Benincasa hispida*, *Cucumis sativus*, *Cucurbita moschata*, *Cucurbita pepo* subsp. *Pepo*, and *Cucurbita maxima*. A 288 bp region within the melon sequence, highly conserved among these cucurbit species, was selected for designing candidate target fragments. *PDS* sequences from the *Cucurbitaceae* family were retrieved from the NCBI GenBank database (the accession number of each sequence can be found in Table S1) and aligned using the software MegAlign 5 [33].

Special restriction enzyme recognition sites were designed by SnapGene 6.0.2 software [34], and are absent in both the infectious clone vector pCambia-ToLCNDV-1.3A and the target fragments. Before the ToLCNDV-VIGS vectors were constructed, two empty vectors (pToLCNDV-SSNK and pToLCNDV-NB) were constructed from pCambia-ToLCNDV-1.3A to ensure equivalence of the lengths of the reorganized *AV1*.

### 2.3. Construction of ToLCNDV-Based VIGS Vectors

#### 2.3.1. Construction of VIGS Vectors with Different Target Fragment Lengths

The empty vector pToLCNDV-SSNK and four *CmPDS* fragments were digested with the restriction endonucleases *Nco*I and *Kpn*I. The reaction mixture contained rCut Smart Buffer (5 μL), *Nco*I (1 μL), *Kpn*I (1 μL), target fragment (16 μL, or empty vector at 8 μL), and nuclease-free water to a final volume of 50 μL. The mixture was incubated at 37 °C for 30 min and heat-inactivated at 75 °C for 10 min. This protocol was also applied to other enzyme combinations. Then, the linearized vector was ligated to each digested target fragment. Four VIGS vectors were constructed, in which the middle region of the *AV1* was substituted with 288 bp, 165 bp, 87 bp, and 48 bp *CmPDS* fragments.

#### 2.3.2. Construction of VIGS Vectors with Different Substitution Areas for the CmPDS Fragment

The optimal size of the target fragment can be determined based on the results from Step 2.3.1. For this purpose, the vector pToLCNDV-NB and the target fragment of optimal size were digested with *Nco*I and *BstE*II, whereas the vector pToLCNDV-SSNK and the optimal target fragment were digested with *Spe*I and *Sma*I. *Sma*I digestion was performed at 25 °C for 15 min, followed by *Spe*I addition and incubation at 37 °C for 30 min. These modifications generated two additional vectors containing target fragments of optimal size with 3′ end substitution and 5′ end substitution, respectively. The VIGS vectors with middle region substitution were previously constructed in Section 2.3.1.

#### 2.3.3. Construction of VIGS Vectors with the Antisense Orientation of CmPDS Fragments

Based on the framework from Section 2.3.1, 165 bp, 87 bp, and 48 bp, the *CmPDS* fragment in the sense orientation was replaced with its antisense counterpart, and VIGS vectors carrying *CmPDS* fragments in the antisense orientation were constructed using the same method.

### 2.4. Agroinfiltration

The *A. tumefaciens* cultures of each VIGS vector, empty vectors (pToLCNDV-SSNK and pToLCNDV-NB) and pCambia-ToLCNDV-1.6B, were adjusted to an optical density (OD_600_) of 1.0. Then, the suspensions of the VIGS vector and the empty vector were mixed with pCambia-ToLCNDV-1.6B suspensions individually at a 1:1 volume ratio and incubated at 28 °C in the dark for 3 h before infiltration into melon plants [35].

Inoculation was performed using the cotyledon injection method [8]. The experiment involved three treatments: VIGS vector inoculation (including the *CmPDS* fragment), a negative control (pToLCNDV-SSNK and pToLCNDV-NB) and a blank control (infiltration buffer; CK). The treatments were set in three repetitions, each including 16 melon plants. After inoculation, the plants were grown immediately under the first 24 h in the dark and then under normal growth conditions with a 16 h light/8 h dark photoperiod [35].

### 2.5. Effects of VIGS Vectors on the Phenotypic Traits of Melon Plants

The number of melon plants displaying photobleaching phenotypes in each VIGS treatment was recorded at 21 days post-inoculation (dpi). 16 plants were observed per trial, with three replicates. Subsequently, the photobleaching severity of leaves from each VIGS-treated plant was graded to determine the photobleaching index, with the following criteria:

Grade 0: No photobleaching symptoms on total leaf area;

Grade 1: photobleaching area accounting for ≤5% of total leaf area;

Grade 3: photobleaching area accounting for >5% and ≤10% of total leaf area;

Grade 5: photobleaching area accounting for 10% and ≤20% of total leaf area;

Grade 7: photobleaching area accounting for >20% and ≤50% of total leaf area;

Grade 9: photobleaching area accounting for >50% of total leaf area.

$$PI=\frac{\sum_{i=0}^{n}\left(Ni\times Gi\right)}{T*\left(Gmax=9\right)}$$

PI: Photobleaching Index; *Ni*: Number of leaves in grade *i*; *Gi*: Grade number corresponding to grade *i*; $\sum_{i=0}^{n}$: Summation over all grades; *T*: Total number of leaves.

Then, the plants in the VIGS treatment group that exhibited the most obvious photobleaching phenotype were selected and grown under the same conditions for further observation of systemic silencing for three months.

### 2.6. Effects of VIGS Vectors on PDS Expression in Melon

Three apical leaves from each treatment group were selected at 9 and 21 dpi, after which total RNA was extracted (FastPure Universal Plant Total RNA Isolation Kit; Vazyme, Nanjing, China) and reverse-transcribed into cDNA (GoScript™ Reverse Transcription System; Promega, Madison, WI, USA), individually. The relative expression level of the *PDS* in melon was determined using reverse transcription quantitative real-time PCR (RT–qPCR) with the primer set CmPDS-qPCR-F (5′-GCAATGCTTGGTGGGCAATC-3′) and CmPDS-qPCR-R (5′-GATTCCTGCAGCTTCCATACC-3′). The expression of the *Actin* (used as an internal reference) of cucurbit plants was determined using RT–qPCR with the primer set CuActin-qPCR-F (5′-CGGGAAATTGTCCGTGACAT-3′) and CuActin-qPCR-R (5′-GATTCCTGCAGCTTC CATACC-3′). RT–qPCR reaction mixture: TB Green Premix ExTaq Mix (10 μL), ROX Reference Dye II (0.4 μL), 10 mmol/L forward and reverse primers (1 μL each), template cDNA (2 μL), and nuclease-free water to a final volume of 20 μL. Amplification program: 95 °C for 30 s; 95 °C for 5 s and 60 °C for 30 s, 40 cycles; 95 °C for 15 s; 60 °C for 1 min. RT–qPCR was performed using a QuantStudio™ 6 Flex Real-Time PCR System (Applied Biosystems, Thermo Fisher Scientific, Waltham, MA, USA). Each reaction contained a negative control with nuclease-free water as the template. Samples collected at 9 dpi and 21 dpi were analyzed in three biological replicates. Relative expression levels were calculated using the 2^−ΔΔCt^ method, and the negative control was set to an arbitrary value (1.0) [8].

## 3. Results

### 3.1. Design of Target Fragments and Construction of Empty Vectors

**Figure 1:**
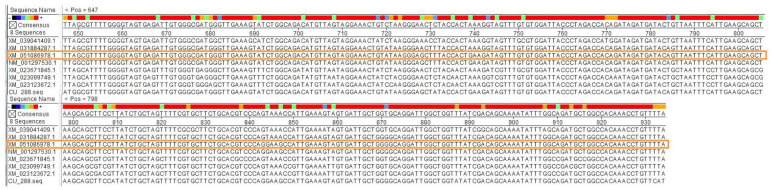
BLAST (NCBI BLAST+ 2.15.0) analysis of 288 bp CDS sequence against *cucurbit* genomes.

A highly conserved 288 bp CDS (spanning 647 to 935 bp) from the *PDS* of melon was selected as a candidate target fragment (Figure 1). Four fragments of different lengths (288 bp, 165 bp, 87 bp, and 48 bp) from the 288 bp CDS above were used as the substitution fragments. Five restriction enzymes (*Spe*I, *Sma*I, *Nco*I, *Kpn*I, and *Bst*EII) were selected, and their enzyme recognition sequences were added to the 5′ and 3′ ends of the substitution fragments. The sequence, restriction enzyme at two ends, and position within *AV1* of each target fragment can be found in Table S2.

**Figure 2:**
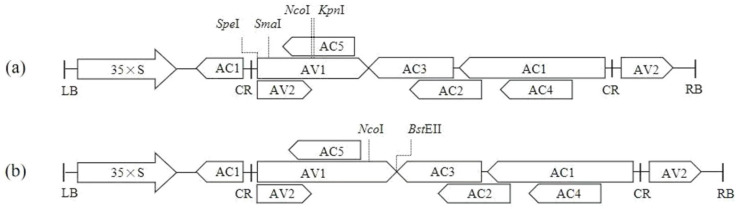
Schematic diagram of the two empty vectors used for constructing the VIGS vectors. (**a**) Vector pToLCNDV-SSNK. (**b**) Vector pToLCNDV-NB. LB: left border; RB: right border; 35xS: CaMV 35S promoter; AV1: coat protein; AV2: pre-coat protein; AC1: replication-associated protein; AC2: transcriptional-activator protein; AC3: replication enhancer protein; AC4: symptom determinant; AC5: viral suppressor of RNA silencing; CR: common region.

The vector pToLCNDV-SSNK, containing *Spe*I, *Sma*I, *Nco*I, and *Kpn*I recognition sites, is designed to insert target fragments (including forward and reverse orientations) into the central and 5′ regions of the *AV1* (Figure 2a), whereas pToLCNDV-NB, containing *Nco*I and *Bst*EII recognition sites, is specifically used to construct VIGS vectors with target fragments positioned at the 3′ ends of the *AV1* (Figure 2b).

All the target fragments and empty vectors sequences were sent to Sangon Biotech (Shanghai) Co., Ltd. (Shanghai, China) for synthesis.

### 3.2. Construction of ToLCNDV-Based VIGS Vectors

**Figure 3:**
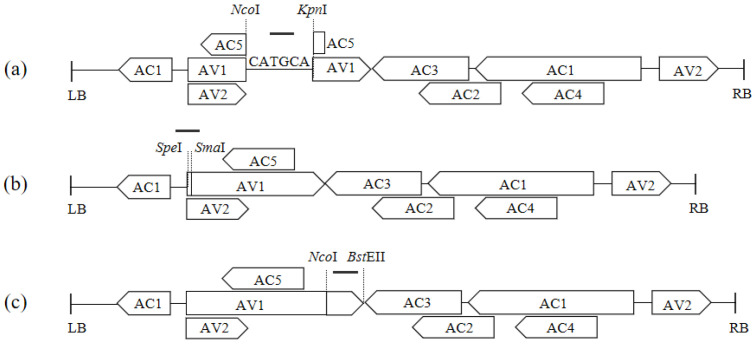
Engineering of VIGS vectors on three different locations of the *AV1* substituted by the target fragment. ‘—’ represents the target fragment. (**a**) Middle substitution. (**b**) 5′ end substitution. (**c**) 3′ end substitution.

In the central region of the viral *AV1* within pToLCNDV-SSNK, a 6 bp sequence was substituted with *CmPDS* fragments in the sense orientation (288 bp, 165 bp, 87 bp, and 48 bp) and antisense orientation (165 bp, 87 bp, and 48 bp). The VIGS vectors used were pToLCNDV-288*CmPDS*-NK, pToLCNDV-165*CmPDS*-NK, pToLCNDV-87*CmPDS*-NK, pToLCNDV-48*CmPDS*-NK, pToLCNDV-165*CmPDS*-RC-NK, pToLCNDV-87*CmPDS*-RC-NK, and pToLCNDV-48*CmPDS*-RC-NK (Figure 3a).

In the 5′ region of the viral *AV1* within pToLCNDV-SSNK, a 30 bp sequence was substituted with a 165 bp *CmPDS* fragment, resulting in the construction of the pToLCNDV-165*CmPDS*-SS vector (Figure 3b).

In the 3′ region of the viral *AV1* within pToLCNDV-NB, a 95 bp sequence was substituted with a 165 bp *CmPDS* fragment, resulting in the construction of the vector pToLCNDV-165*CmPDS*-NB (Figure 3c). The final maps can be found in Figure S2.

### 3.3. Effects of VIGS Vectors on Melon Leaf Photobleaching

**Figure 4:**
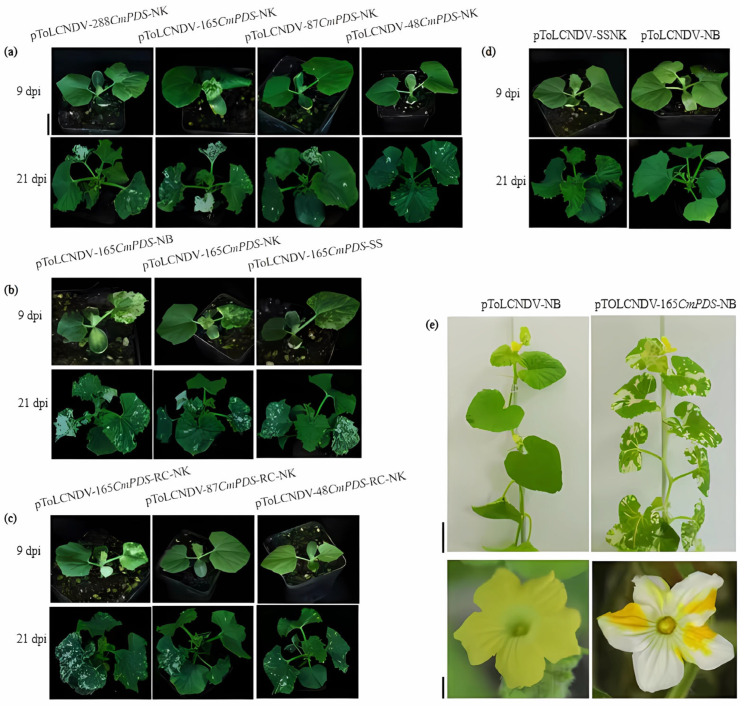
Phenotypic alterations in melon plants after inoculation. (**a**) Leaf photobleaching in response to VIGS treatments with varying target fragment lengths. (**b**) Leaf photobleaching in the VIGS treatments with the 165 bp *CmPDS* fragment substituting different areas of the *AV1*. (**c**) Leaf photobleaching in the VIGS treatments with the *CmPDS* fragment inserted in the antisense orientation of different lengths. (**d**) Leaf phenotype in the control treatments. (**e**) Systemic silencing phenotype in newly emerged leaves and flowers after pToLCNDV-165*CmPDS*-NB treatment. Bar = 2 cm, 5 cm, and 1 cm, from bottom to top.

Melon plant phenotypes were photographed at 9, 21, and 90 dpi. At 9 dpi, melon plants with 165 bp target fragment treatment (pToLCNDV-165*CmPDS*-NK, pToLCNDV-165*CmPDS*-NB and pToLCNDV-165*CmPDS*-RC-NK) exhibited a marked photobleaching phenotype, whereas those subjected to other VIGS treatments did not present the same phenotypes. At 21 dpi, all the VIGS treatments exhibited a marked photobleaching phenotype. No visible photobleaching occurred in the plants of pToLCNDV-SSNK and pToLCNDV-NB control, respectively (Figure 4d).

Among the VIGS treatments with different sizes of target fragments, the vector pToLCNDV-165*CmPDS*-NK resulted in the highest incidence of systemic silencing plants and the highest photobleaching index (Table 1, Figure 4a). Furthermore, compared to the pToLCNDV-165*CmPDS*-SS with 5′ end substitution, the pToLCNDV-165*CmPDS*-NB treatment with the 3′ end substitution also achieved higher systemic silencing incidence of 100% and photobleaching index (Table 1, Figure 4b). Antisense- and sense-targeted VIGS vectors resulted in consistent systemic silencing incidence and photobleaching index without apparently statistics difference (Table 1; Figure 4a,c).

To investigate the long-term gene silencing stability of the ToLCNDV-based VIGS vector during extended growth periods, we cultured pToLCNDV-165*CmPDS*-NB-treated plants for three months. The newly emerged leaves and flowers of these plants also displayed photobleaching phenotypes (Figure 4e).

### 3.4. Influence of VIGS Vectors on PDS Expression in Melon

#### 3.4.1. Silencing Effects of the Length of the CmPDS Fragment

**Figure 5:**
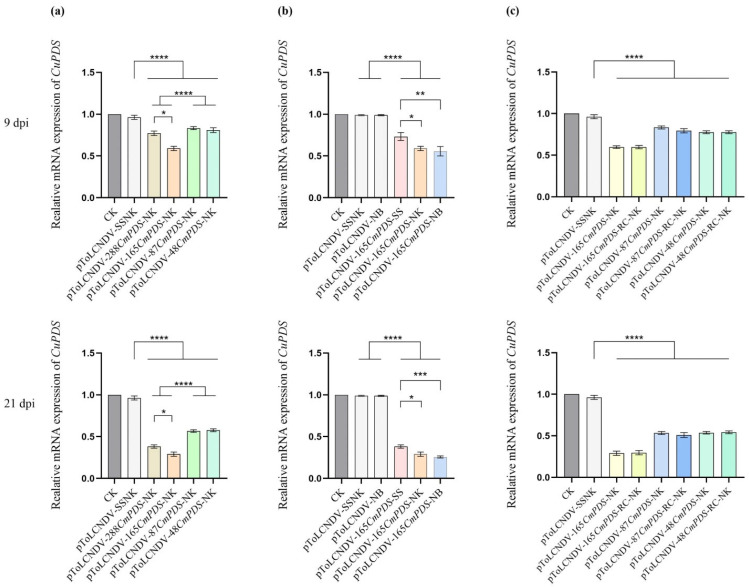
RT–qPCR analysis of *PDS* expression in melon plants after VIGS treatment. Three technical replicates were performed for each individual sample from both 9 dpi and 21 dpi. Significant differences between VIGS treatments are indicated as follows: *: *p* < 0.05, **: *p* < 0.01, ***: *p* < 0.001, ****: *p* < 0.0001 by ANOVA post hoc Test (Tukey). The error bars indicate the SDs. (**a**) Silencing efficiency of VIGS vectors carrying *CmPDS* fragments of different sizes. (**b**) Silencing efficiency of VIGS vectors with 165 bp *CmPDS* fragments substituting different areas of the *AV1*. (**c**) Silencing efficiency of VIGS vectors with 165 bp, 87 bp, and 48 bp *CmPDS* fragments in the sense and antisense orientation.

To investigate the impact of target fragment length on gene silencing efficiency, RT–qPCR was performed to analyze the mRNA accumulation of the *PDS*. The results revealed that all the VIGS treatments resulted in significantly lower *PDS* expression at 9 and 21 dpi (*p* < 0.0001). In addition, compared to the other VIGS treatments, the pToLCNDV-165*CmPDS*-NK treatment resulted in the most significant reduction in *PDS* expression at both 9 and 21 dpi (Figure 5a). Compared to the pToLCNDV-87*CmPDS*-NK and pToLCNDV-48*CmPDS*-NK treatments, the pToLCNDV-288*CmPDS*-NK treatment also resulted in significantly lower *PDS* expression (*p* < 0.05). Meanwhile, the expressions between pToLCNDV-87*CmPDS*-NK and pToLCNDV-48*CmPDS*-NK treatments did not significantly differ. These findings suggested that the 165 bp target fragment achieved maximum silencing efficiency.

#### 3.4.2. Silencing Effects of the Substitution Area of the CmPDS Fragment

To further evaluate the influence of the area of the target fragment within the *AV1* on VIGS efficiency, RNA samples from the pToLCNDV-165*CmPDS*-NB, pToLCNDV-165*CmPDS*-SS, and pToLCNDV-165*CmPDS*-NK treatments were analyzed (Figure 5b). The results revealed that compared to those in the pToLCNDV-165*CmPDS*-SS treatment group, the amounts in the pToLCNDV-165*CmPDS*-NB and pToLCNDV-165*CmPDS*-NK treatments were significantly lower at 9 dpi (*p* < 0.01 and *p* < 0.05, respectively). Moreover, the differential expression levels of *PDS* between the pToLCNDV-165*CmPDS*-NB and pToLCNDV-165*CmPDS*-SS treatments further increased at 21 dpi (*p* < 0.001). These findings confirmed that compared to 5′ end insertion, the VIGS system with middle and 3′ end insertion resulted in greater silencing efficiency.

#### 3.4.3. Silencing Effects of CmPDS Fragments in the Antisense Orientation

The effects of target fragment sequence orientation on silencing efficiency were also evaluated. The results revealed that *PDS* expression levels in the antisense-targeted VIGS vectors (pToLCNDV-165*CmPDS*-RC-NK, pToLCNDV-87*CmPDS*-RC-NK, and pToLCNDV-48*CmPDS*-RC-NK) did not significantly differ from those in the corresponding sense-targeted VIGS vectors at either 9 or 21 dpi (Figure 5c). The results demonstrated that the sequence orientation of the target fragment did not significantly affect the gene silencing efficiency of the ToLCNDV-based VIGS system.

## 4. Discussion

In this study, we successfully engineered ToLCNDV as a VIGS vector and effectively silenced the *PDS* in melon. We systematically investigated the impact of the insertion length, position within the *AV1*, and sequence orientation of the target fragment on gene silencing. Our results demonstrated that the target fragment insertion length was the critical factor for the ToLCNDV-VIGS system and further revealed that the ToLCNDV-based VIGS system is capable of sustainably inducing gene silencing in melon. To the best of our knowledge, this is the first application of ToLCNDV as a VIGS vector for endogenous gene silencing in melon.

The insertion of target gene fragments of excessive length in viral vectors tends to destabilize the viral genome, impairing infectivity and increasing off-target effects. Conversely, overly short inserts can compromise vector stability and thereby reduce silencing efficiency [36,37,38]. However, precisely designed short RNAs (such as sgRNAs) have been demonstrated to effectively target critical regions of genes, enabling highly efficient gene silencing [39]. Previous research has successfully engineered ACMV and AbMV into a VIGS vector, demonstrating that the DNA-A component lacking the *AV1* can achieve systemic spread in planta when it is co-administered with DNA-B [13,14]. We initially positioned the exogenous gene within the central region of the *AV1* to construct VIGS vectors harboring target fragments of varying lengths. Our results showed that VIGS vectors with 288, 165, 87, and 48 bp target fragments efficiently silenced the *PDS* (reducing *PDS* transcript levels by 50–75%), with 165 bp achieving the greatest silencing efficiency—slightly deviating from the reported optimal range of 200–350 bp [40,41,42,43]. For example, employing barley striped mosaic virus (BSMV) as a VIGS vector to silence the *PDS* revealed that the optimal insert lengths were approximately 200 bp in barley [40] and 275 bp in wheat [41]. In TRV-based VIGS studies, the insertion of a 255 bp *JrPOR* (protochlorophyllide reductase) fragment elicited the most pronounced photobleaching phenotype in *walnut* [42], whereas the introduction of a 301 bp cDNA fragment enabled efficient silencing of the *PDS* in soybean [43]. However, when TYLCCNV was engineered as a VIGS vector to silence the *GFP* and *PDS* in 16c tobacco, an optimal insert length of 170 bp was identified [15], which is consistent with our findings.

The position of the target fragment within the *AV1* also influences the viability, stability, infectivity, and silencing efficiency of the VIGS vector [8]. Next, we repositioned the exogenous gene at both the 5′ and 3′ ends of the *AV1* to construct VIGS vectors. Our work revealed that compared to the 5′ end substitution, the 3′ end and mid-region substitutions in this study resulted in significantly higher silencing efficiency. These results indicate that positioning the insertion site proximal to the 3′ end of the *AV1* significantly enhances viral replication and accumulation efficiency. This may be closely tied to the gene architecture and the distribution of functional elements. The 3′ non-transcribed region (UTR) often contains regulatory elements, and the insertion of target genes into this region minimizes interference with viral replication and systemic movement [44]. In contrast, insertions near the 5′ end (coding region) may disrupt the translation of critical viral proteins, such as promoters, thereby reducing viral replication capacity and silencing efficiency [45]. For example, the same 3′ UTR of the coat protein-encoding gene insertion strategy has been proven to efficiently express greater amounts of foreign genes when citrus tristeza virus (CTV) and lettuce infectious yellows virus are used [46,47]. In this study, the central region insertion within the *AV1* also affected the *AC5*, which is associated with gene silencing suppression [11], while the 5′ end insertion influenced the *AV2* (encoding the movement protein). This also explains the higher silencing efficiency observed in VIGS vectors constructed with central-region insertion than in those constructed with 5′ end insertion.

The sequence orientation of the target gene may impact the silencing efficiency of the VIGS vectors. Some reports have demonstrated that antisense inserts can achieve greater silencing than those with sense insertion can when the bean pod mottle virus (BPMV) system [48], brome mosaic virus (BMV) system [49], CTV system [50], and rice tungro bacilliform virus (RTBV) system are used [51], whereas other studies have suggested that silencing efficiency is not influenced by the insert orientation when the barley stripe mosaic virus (BSMV) system [40], tobacco mosaic virus (TMV) system [52], cabbage leaf curl virus (CaLCuV) system [53], or TRV-VIGS system are used [54]. To further study the impact of the insertion orientation of the ToLCNDV-VIGS system on silencing efficacy, we inserted 165, 87, and 48 bp antisense target fragments within the central region of the *AV1*. Our results suggest that compared to sense insertions, antisense insertions resulted in equivalent silencing efficiency.

## 5. Conclusions

In this report, the conditions for efficient virus-mediated gene silencing in melon were optimized using the ToLCNDV-VIGS system. To achieve the most efficient gene silencing, the best combination is to insert a 165 bp target fragment into the 3′ end of the *AV1* within ToLCNDV. Melon plants with this VIGS treatment achieved the highest photobleaching incidence and index, along with the lowest *PDS* expression level. These findings will be highly meaningful when the gene functions of melon and other cucurbits crops, particularly those with limited genetic transformation tools, are applied in the future.

## Figures and Tables

**Table 1:** Photobleaching percentage and index of melon plants whose expression was systemically silenced. The data were derived from three replicates.

VIGS Systems (with pCambia-ToLCNDV-1.6B)	Systemically Silenced Plants/Total Infiltrated Plants	Incidence of Silenced Plants in Average (%)	Photobleaching Index in Average
pToLCNDV-288*CmPDS*-NK	14/16, 14/16, 15/16	89.58 abc	43.50 c
pToLCNDV-165*CmPDS*-NK	15/16, 16/16, 16/16	97.92 a	61.75 b
pToLCNDV-87*CmPDS*-NK	13/16, 14/16, 13/16	83.33 bc	29.34 d
pToLCNDV-48*CmPDS*-NK	14/16, 13/16, 12/16	81.25 c	25.67 d
pToLCNDV-165*CmPDS*-NB	16/16, 16/16, 16/16	100 a	76.50 a
pToLCNDV-165*CmPDS*-SS	13/16, 12/16, 13/16	79.17 c	48.13 c
pToLCNDV-165*CmPDS*-RC-NK	15/16, 15/16, 16/16	95.83 ab	60.58 b
pToLCNDV-87*CmPDS*-RC-NK	14/16, 12/16, 13/16	81.25 c	27.91 d
pToLCNDV-48*CmPDS*-RC-NK	13/16, 13/16, 14/16	83.33 bc	26.29 d

Note: Incidence refers to the percentage of plants that show photobleaching. Significant differences between VIGS Systems are indicated in lowercase letters by ANOVA post hoc Test (Duncan) at alpha = 0.01.

## Data Availability

The original contributions presented in this study are included in the article. Further inquiries can be directed to the corresponding authors.

## Supplementary Materials

The following supporting information can be downloaded at: https://www.mdpi.com/article/10.3390/pathogens14121269/s1, Figure S1: Schematic diagram of pCambia-ToLCNDV-1.3A and pCambia-ToLCNDV-1.6B; Figure S2: Schematic diagram of 5′ end, middle region substitution and 3′ end substitution. Take the insertion of a 165 bp target fragment as an example; Table S1: GenBank accession numbers of PDS sequences used for candidate target fragments screening; Table S2: Sequence, restriction enzyme at two ends, and positions of target fragments within *AV1* used to construct VIGS vectors.
