# Supplementary material for: A Begomovirus solanumdelhiense Vector for Virus-Induced Gene Silencing in Melon
Source: Pathogens. 2025 Dec 10;14(12):1269. doi: 10.3390/pathogens14121269 (PMC12735790; doi:10.3390/pathogens14121269)
Supplement: Supplementary file 1 [file pathogens-14-01269-s001.zip › pathogens-3995392-supplementary.pdf]

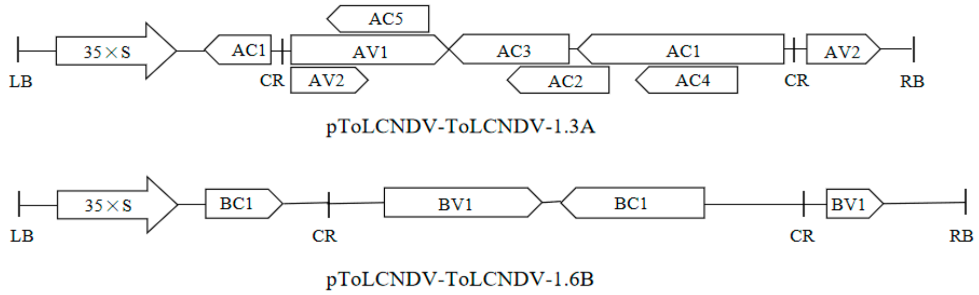

Figure S1. Schematic diagram of pCambia-ToLCNDV-1.3A and pCambia-ToLCNDV-1.6B. LB: left border; RB: right border; 35xS: CaMV 35S promoter; AV1: coat protein; AV2: pre-coat protein; AC1: replication-associated protein; AC2: transcriptional-activator protein; AC3: replication enhancer protein; AC4: symptom determinant; AC5: viral suppressor of RNA silencing; BV1: Movement protein; BC1: nuclear shuttle protein; CR: common region.

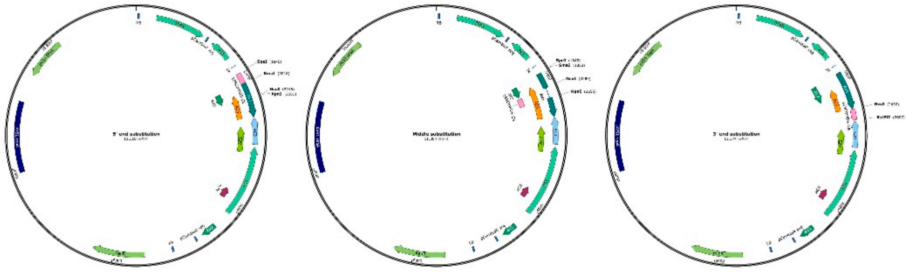

Figure S2. Schematic diagram of 5' end, middle region substitution and 3' end substitution. Take the insertion of a 165 bp target fragment as an example.

Table S1. GenBank accession numbers of PDS sequences used for candidate target fragments screening.

GenBank accession number	species
XM_039041409.1	*Benincasa hispida*
XM_031884287.1	*Cucumis sativus*
XM_051086978.1	*Cucumis melo*
NM_001297530.1	*Cucumis melo*
XM _023671845.1	*Cucurbita pepo subsp. pepo*
XM_023099749.1	*Cucurbita moschata*
XM_023123672.1	*Cucurbita maxima*

Table S2. Sequence, restriction enzyme at two ends, and positions of target fragments within *AV1* used to construct VIGS vectors

Target fragment	Sequences(5'→3')	Restriction Enzymes at the 5' end	Restriction Enzymes at the 3' end	Inserting position Within *AV1*
288*CmPDS*-NK	atggcgttttggggtagtgagattgtgggcgatgggttgaaagtatctggcagacatgttagtaggaaactgtataagggagctataccactgaagatagtttgtgtggattaccctagaccacagatagatgatacagttaatttcattgaagcagcttccatatctgctagttttcgtgcttctgcacgtcccaggaagccattgaaagtagtgattgctggggcaggattggctggtatatcgacagcaaaatatttggcagatgctggccacaaacctgttcat	*Nco*I	*Kpn*I	middle
165*CmPDS*-NK	atggcgttttggggtagtgagattgtgggcgatgggttgaaagtatctggcagacatgttagtaggaaactgtataagggagctataccactgaagatagtttgtgtggattaccctagaccacagatagatgatacagttaatttcattgaagcagcttccata	*Nco*I	*Kpn*I	middle
165*CmPDS*-NB		*Nco*I	*Bst*EII	3' end
165*CmPDS*-SS		*Spe*I	*Sma*I	5' end
165*CmPDS*-RC-NK	tatggaagctgcttcaatgaaattaactgtatcatctatctgtggtctagggtaatccacacaaactatcttcagtggtatagctcccttatacagtttcctactaacatgtctgccagatactttcaacccatcgcccacaatctcactaccccaaaacgccat	*Nco*I	*Kpn*I	middle
87*CmPDS*-NK	gcgttttggggtagtgagattgtgggcgatgggttgaaagtatctggcagacatgttagtaggaaactgtataagggagctata	*Nco*I	*Kpn*I	middle
48*CmPDS*-NK	gcgttttggggtagtgagattgtgggcgatgggttgaaagtatctggc	*Nco*I	*Kpn*I	middle
87*CmPDS*-RC-NK	tatagctcccttatacagtttcctactaacatgtctgccagatactttcaacccatcgcccacaatctcactaccccaaaacgc	*Nco*I	*Kpn*I	middle
48*CmPDS*-RC-NK	gccagatactttcaacccatcgcccacaatctcactaccccaaaacgc	*Nco*I	*Kpn*I	middle
